# Volume and Tempo: Cortical Excitability and Trial-to-Trial Consistency of Auditory Responses Distinguish Psychosis Biotypes

**DOI:** 10.21203/rs.3.rs-9336633/v1

**Published:** 2026-05-19

**Authors:** John Greco, David Parker, Elena Ivleva, Godfrey Pearlson, Sarah Keedy, Matcheri S. Keshavan, Elliot Gershon, Carol Tamminga, Scot Hill, Jennifer McDowell, Brett Clementz

**Affiliations:** University of Georgia; University of Texas Southwestern Medical Center; Department of Psychiatry, Yale University; University of Chicago; Harvard Medical School; The University of Chicago; University of Texas southwestern medical center; Rosalind Franklin University of Medicine and Science; University of Georgia

## Abstract

ERPs average neural responses to stimuli across trials. This accentuates consistent activity over time but obscures differences in response strength and phase consistency from trial to trial. The Bipolar-Schizophrenia Network for Intermediate Phenotypes (B-SNIP) identified three transdiagnostic psychosis Biotypes (BT1, BT2, BT3) that differ in cortical excitability and temporal coherence of neural responses. Single-trial power (STP) and inter-trial coherence (ITC) were measured across 3–55 Hz in auditory paired-stimuli and oddball tasks using a large multi-site cohort (n = 2 373). One-way ANOVAs over a continuous time-frequency epoch with FDR and cluster correction identified group differences, resulting in 15 significant STP and ITC features. Linear discriminant analysis of these features identified the dimensions that maximally separated Biotypes. The STP differences covered the entire trial epoch, showing the strongest alterations in the beta band (18–32 Hz), graded BT2 > > BT3 > HC > > BT1. The ITC differences were seen at specific time points primarily in low-frequency bands (3–17 Hz), graded HC > BT3 > BT1 > BT2. These dimensions were orthogonal and did not mirror ERP amplitudes across Biotypes. Biotypes were better distinguished by STP and ITC (Euclidean distances 0.9–2.2) than by DSM diagnosis (0.10–0.45). DSM groups alone did not benefit from this breakdown. Cortical excitability and phase precision are unique neurophysiological targets for the etiological investigation of psychosis Biotypes, which traditional ERP averaging obscures.

## Introduction

Serious psychiatric conditions like schizophrenia, schizoaffective disorder, and bipolar disorder with psychosis are currently diagnosed from clinical characteristics. These categories mask substantial variability in brain structure and function. Patients sharing the same label can have different neural profiles, while patients with different labels can share neural characteristics. Use of such categories may blur understanding of how symptoms arise and impede the development of targeted treatments. A strategy including biomarkers aims to carve neurobiologically cohesive subgroups that may meaningfully refine psychosis diagnoses [5].

The Bipolar-Schizophrenia Network for Intermediate Phenotypes (B-SNIP) used one such strategy. Laboratory measures from EEG/ERP, ocular motor, and cognitive domains were used in numerical taxonomy, and revealed three neurobiologically distinctive psychosis Biotypes [1]. The patterns replicated and cross-validated in an independent cohort [3]. B-SNIP Biotypes have been refined and fortified [10]. Multiple external validations demonstrate that psychosis Biotypes better account for results at other levels of analysis than DSM psychosis diagnoses [5, 11, 12]. A diagnostic algorithm is available that yields Biotypes using limited clinical [4] and cognitive evaluations [6].

B-SNIP Biotypes capture neurobiological patterns that differ systematically in cognitive performance, background neural activity, and sensory responsiveness. BT1 has deficient cognitive performance, low background brain activity, and diminished neural responses to salient stimuli in grand-average ERPs, sluggish sensory processing, and the overall lowest brain volumes [12, 13, 14]. Alternatively, BT2 has the worst cognitive performance, accentuated background brain activity, relatively intact neural responses to salient stimuli in grand-average ERPs, and the worst sensory-motor inhibition. Both BT1 and BT2 have compromised signal-to-noise ratios, but for different reasons (a low numerator in BT1 but a high denominator in BT2). BT3 is closest to healthy on most indicators of brain function and structure, but with modest deviations in registering stimulus salience, accompanied by the fastest visual orienting, and subtle structural deviations of lateral thalamic nuclei [3, 10, 12, 14].

Measures of brain physiology are essential Biotype-defining characteristics. Two auditory tasks (paired-stimuli and oddball) capture psychosis-related neural dysfunction in event-related potentials (ERPs). In combination, they probe the adequacy of neural responses to salient stimuli, plus recovery from and preparation for auditory stimulation [3, 10, 15, 16], and context updating in working memory [17, 18].

Traditional ERP analyses emphasize temporally consistent activity by averaging many trials keyed to a single time point of interest (like the onset of repeating auditory stimuli). ERPs miss neural activity that varies temporally across trials relative to that event. Analyses of individual trials address this imbalance by quantifying neural response strength regardless of temporal consistency (single-trial power, or STP) and neural response consistency regardless of strength (inter-trial phase coherence, or ITC). In this paper, we probe auditory response patterns of psychosis Biotypes via STP and ITC.

Prior auditory processing investigations of psychosis illustrate deviations of both STP and ITC, including hints of frequency band-specific dysregulation [19]. During paired-stimuli paradigms, schizophrenia cases show poorly coordinated responses to the first stimulus (S1) and frequency-specific event-related desynchronization before S2 [15, 21]. The P300 is shaped by the coordination and strength of delta-theta activity [18]. In both schizophrenia and bipolar disorder, poor delta-theta phase consistency may cause P300 deviations [22]. Smaller P300 amplitudes in psychosis are also linked to reduced delta-theta phase locking and reduced neural power [23] Neural oscillatory disturbances during auditory processing also may involve higher frequencies in the beta and gamma ranges [24, 25, 26, 27].

Neurophysiologically distinctive psychosis subgroups may more accurately capture the heterogeneity of neural responses observed across clinical psychosis diagnoses. Neural activity patterns differentiating B-SNIP psychosis Biotypes replicate across auditory EEG/ERP measures [3, 8, 9, 10, 15, 20, 28, 29]. To complement and refine those findings, this paper describes single-trial STP and ITC patterns during auditory paired-stimuli and oddball paradigms across psychosis Biotypes. We predicted that (i) STP would emerge as an independent response magnitude dimension on which BT1 and BT2 would be at opposite ends of the spectrum, (ii) ITC would discriminate Biotype groups on a separate temporal precision dimension, (iii) a combination of these dimensions would more effectively discriminate Biotype groups than either measure alone, and (iv) DSM psychosis diagnoses would not benefit from separating response strength from response consistency.

## Methods

### Participants and grouping

We analyzed data from B-SNIP1, B-SNIP2, and PARDIP under IRB-approved protocols. All participants provided informed consent. Participants were recruited across multiple sites; psychosis cases met DSM-IV-TR criteria for schizophrenia (n = 677), schizoaffective disorder (n = 519), or bipolar I disorder with psychosis (n = 488). Healthy persons were recruited to be demographically comparable but without personal or first-degree family history of major psychiatric conditions [30]. Key exclusions included neurological illness, significant head injury, and recent substance dependence; these safeguards minimized non-psychosis sources of EEG variance [3, 10].

Psychosis participants were assigned to Biotypes (BT1, BT2, BT3) using previously described multivariate procedures [10]. Briefly, a set of 11 neurobiological composite variables spanning cognitive performance, behavioral neurology, and electrophysiology were entered into an analysis pipeline that reproducibly identified three neurobiological distinctive subgroups [5, 10]. The Biotype solution replicated, cross-validated, and externally validated across B-SNIP phases with similar subgroup characteristics, supporting its stability. For the subset with complete EEG on both auditory paradigms (oddball and paired stimuli) the dataset contains n = 2 373 participants with the following group counts: BT1 = 515, BT2 = 512, BT3 = 538, HC = 808. See Supplementary Tables 2 (demographics) and 3 (medications) for descriptive details.

## EEG acquisition

EEG was continuously recorded from 64 Ag/AgCl sensors (impedance < 10KΩ; Quik-Cap, Compumedics Neuroscan, El Paso, TX), positioned according to the standard 10–10 EEG system plus mastoids and CB1/2 locations to provide sampling below the cantho-meatal line, with nose reference and forehead ground. Recordings were amplified (×12 500) and digitized (1000 Hz) using Neuroscan Acquire and Synamps2 recording systems [10].

## Tasks

### Common Recording Procedures

Data collection and pre-processing of EEG/ERPs followed procedures established in Parker et al. (2020, 2021, 2025) and were applied to all subjects in this paper. Minor modifications from Clementz et al. (2016, 2022) ensured standardized data quality control between projects [10]. Changes to the scoring procedures were primarily to frequency domain quantification and were made to simplify the analysis steps and improve the ease of replication and verification by other laboratories.

Recording conditions were equivalent and stimulus presentation and recording equipment identical across sites. Participants were seated in a sound and electrically shielded booth (ambient sound = 61–63 dB; luminance = 0.11–0.12 foot-candles). Participants who were smokers refrained from smoking 1 hour prior to testing [10].

## Auditory Paired Stimuli

Subjects passively listened to 120–150 (B-SNIP1: 150, PARDIP/B-SNIP2 Sample: 120) binaural broadband auditory click pairs (4 msec duration at 75 dB sound pressure level; 500 msec inter-click interval) occurring an average of every 9.5 sec (9–10 sec inter-pair interval) and delivered through headphones. Data were segmented into epochs from 200 msec before to 880 msec after click-pair onset. Epochs containing activity ± 75 μV were eliminated.

### Auditory oddball

Stimuli were 567 standard (1000 Hz) and 100 target (1500 Hz) tones presented in pseudorandom order (1300 msec inter-trial interval). Subjects were asked to press a button when a target was detected. Data were segmented into epochs from 200 msec before to 600 msec after stimulus. Epochs containing activity ± 75 μV were eliminated.

## EEG preprocessing

Raw EEG data were inspected for bad sensors and artifacts. Bad sensors were interpolated (< 5% of all sensors for any subject) using spherical splines (BESA 5.3; MEGIS Software, Grafelfing, Germany). Data were converted to an average reference and digitally band-pass filtered from 0.5–55 Hz (zero phase filter; rolloff: 6 and 48 dB/octave, respectively). Blink and cardiac artifacts identified using independent components analysis were removed (EEGLAB 9.0) [10].

## Time-frequency decomposition and feature derivation

Following Ethridge et al. (2015) and Hamm et al. (2014), single-trial power (STP) and inter-trial coherence (ITC) were computed using complex Morlet wavelets implemented in EEGLAB [31, 32]. Frequencies spanned 3–55 Hz with approximately 50 logarithmically spaced bins, using 1 to 8 cycles increasing linearly with frequency to balance temporal and spectral resolution.

Single-trial power was quantified as raw power, expressed as the magnitude of oscillatory power at each time-frequency bin, reflecting the absolute amplitude of ongoing neural activity. Inter-trial coherence was computed as the magnitude of the mean unit phase vector across trials (range 0–1), indexing the temporal consistency of phase-locked responses [7, 9]. STP and ITC were treated as separable physiological dimensions, reflecting differences in response strength and temporal precision, respectively.

**Figure 1:**
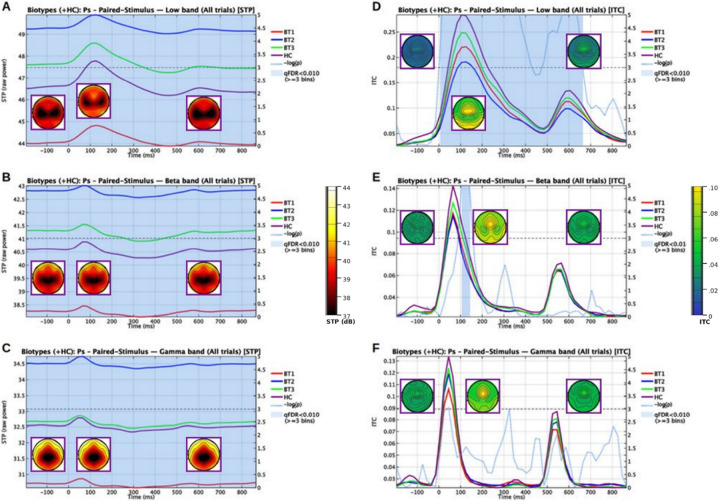
Paired-Stimulus raw single-trial power (Left Column, A-C) and inter-trial coherence (Right Column, D-F). Group-average waveforms (~3–55 Hz) for Biotypes (BT1–BT3) and healthy participants (HC) across the −200 to 880 ms epoch time-locked to S1 onset. Shaded regions indicate time bins surviving cluster-corrected ANOVA thresholds (qFDR < .01, ≥3 contiguous bins); the dotted trace shows −log10(p) values across time. Whole-scalp averaging across 64 electrodes was employed to maximize signal reliability and isolate global response dimensions. Topographic maps (HC) at −200 to 0, 0 to 200, and 500 to 880 ms illustrate the scalp distributions underlying the averaged signal; biotype-specific topographies are provided in the Supplementary Materials.

**Figure 2:**
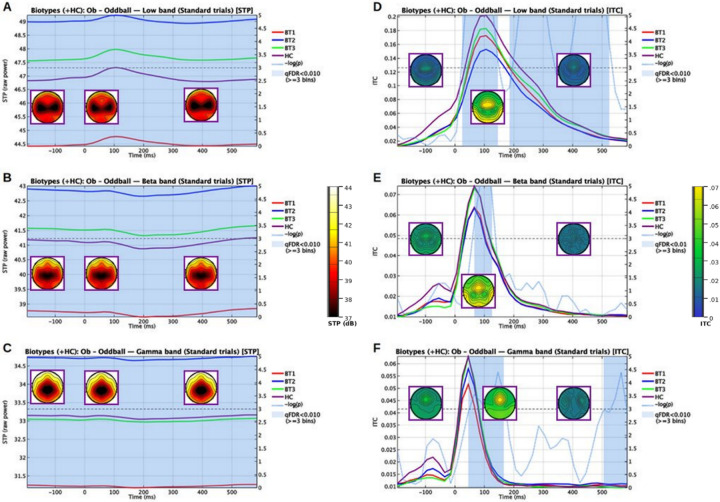
Oddball Standard trials raw single-trial power (Left Column, A-C) and inter-trial coherence (Right Column, D-F). Group-average waveforms (~3–55 Hz) for Biotypes (BT1–BT3) and healthy participants (HC) across the −200 to 600 ms epoch. Shaded regions indicate time bins surviving cluster-corrected ANOVA thresholds (qFDR < .01, ≥3 contiguous bins); the dotted trace shows −log10(p) values across time. Whole-scalp averaging across 64 electrodes was employed to maximize signal reliability and isolate global response dimensions. Topographic maps (HC) at −200 to 0, 0 to 150, and 350 to 600 ms illustrate the scalp distributions underlying the averaged signal; biotype-specific topographies are provided in the Supplementary Materials.

For descriptive visualization and secondary feature summarization, time-frequency estimates were grouped into empirically derived frequency ranges: Low (3–17 Hz), Beta (18–32 Hz), and Gamma (33–55 Hz), consistent with prior B-SNIP analyses of auditory processing [8, 9, 10, 15, 20]. Prior to group-level analyses, STP and ITC estimates were averaged across all 64 sensors at each time-frequency bin. To evaluate the accuracy of cross-sensor values, intraclass correlations was computed for each 20 ms bin across all 64 sensors, separately for each paradigm, trial type, and frequency band. As can be seen in Figs. 1 and 2, STP and ITC were stationary over time within every task, although the spatial patterns differed by frequency. Rather than creating spatially reduced time courses within each task and frequency, we investigated the reliability of all 64 sensors for STP and ITC using ICCs (see Supplementary Results). Reliability can be considered a guide for the number measurements needed to capture an individuals’ true value for some parameter. Falconer and Mackay’s [34] formula was used to calculate the gain from 64-sensor measurements to estimate a participant’s true performance (which would be a value of 1.0). Single-sensor ICCs were reasonable (see Supplementary Results) and corresponding 64-sensor gain estimates were consistently high across bins for STP, r(64) = .983–.996, and ITC, r(64) = .936–.986 (see Supplementary Table 1 for details). This means that averaging the 64-sensor data provided a substantial gain in measurement accuracy for every participant.

### Statistical analysis

Primary group effects were tested using ANOVAs computed at each time-frequency bin for each task and trial type, yielding statistical maps that localized group differences across time and frequency. Multiple-comparison control combined two sequential steps. First, false discovery rate correction (q = 0.01) was applied across all time-frequency bins to identify individually significant voxels while controlling the expected proportion of false positives. Second, to account for the nonindependence of adjacent time-frequency bins and further guard against isolated false positives, a cluster-based permutation test was applied to the FDR-surviving voxels [15, 33]. Clusters were defined as contiguous regions of ≥ 3 adjacent time-frequency bins and assessed against a null distribution generated from 5 000 shuffles with a cluster-level significance threshold of α = 0.01. Only regions meeting both FDR and cluster-level criteria were considered interpretable group differences. These analyses were designed to localize when and at which frequencies Biotype differences emerged and to distinguish differences in response strength versus temporal precision. Glass effect sizes (Δ) in relation to healthy values were also used to compare groups.

### Discriminant analysis

To organize and ease interpretation of multivariate patterns across statistically significant effects, linear discriminant analysis (LDA) was applied using SPSS Statistics version 30.0.0.0 (Armonk, NY: IBM Corp.). Included features consisted of values extracted from significant ANOVAs. Interpretation of the linear discriminant analyses focused on identifying patterns of time-frequency features most strongly differentiated groups. To do so, we examined the features that defined significant discriminant functions via the structure matrices. This approach allowed for efficiently determining patterns of neural magnitude and temporal precision features that maximized group separations.

## Results

### Paired-stimuli results

Responses in the paired-stimuli task were evaluated relative to S1 across the full − 200 to + 880 ms epoch using continuous time-frequency ANOVAs. Group effects were examined separately for single-trial power (STP) and inter-trial coherence (ITC) across Low (3–17 Hz), Beta (18–32 Hz), and Gamma (33–55 Hz). Effects were summarized descriptively using group means, standard deviations, and effect sizes from the statistically significant time-frequency regions. For DSM diagnosis comparisons, see DSM Supplementary Results.

### Single-trial power (STP; response strength)

#### (1) Low-frequency STP (-200 to 880 ms).

BT2 exhibited the highest power (Δ = 0.82), and BT1 the lowest (Δ = −0.78). BT3 (Δ = 0.32) and healthy participants showed intermediate values (Table 1). This graded ordering (BT2 > BT3 > HC > BT1) was evident across the full epoch (Fig. 1A).

#### (2) Beta STP (-200 to 880 ms).

The largest group separations were observed in the beta band. BT2 exhibited the highest power (Δ = 0.88), and BT1 the lowest (Δ = −0.91). BT3 (Δ = 0.27) and healthy participants showed intermediate values (Table 1). This graded ordering (BT2 > BT3 > HC > BT1) was also evident across the full epoch (Fig. 1B).

#### (3) Gamma STP (-200 to 880 ms).

Gamma-band separations were smaller than those observed in the beta band but followed a similar pattern. BT2 showed the highest power (Δ = 0.70), and BT1 the lowest (Δ = −0.64). BT3 (Δ = 0.04) and healthy participants, who did not significantly differ, exhibited intermediate values (Table 1). This ordering (BT2 > (BT3 = HC) > BT1) was evident across the full epoch (Fig. 1C).

### Inter-trial coherence (ITC; temporal precision)

#### (4) Low-frequency ITC (5 to 665 ms).

In contrast to STP, low-frequency ITC effects were temporally circumscribed rather than continuous across the epoch. Significant group differences did not include the baseline. For low-frequency ITC (Table 1), healthy participants exhibited the highest phase consistency, whereas BT2 had the lowest ITC (Δ =−0.71). BT1 (Δ =−0.49) and BT3 (Δ =−0.27) were intermediate, producing an ordering of HC > BT3 > BT1 > BT2 (Fig. 1D).

#### Beta ITC.

Beta-band ITC did not exhibit statistically significant group differences across the paired-stimuli epoch (Fig. 1E).

#### Gamma ITC.

Gamma-band ITC did not exhibit statistically significant group differences across the paired-stimuli epoch (Fig. 1F).

### Oddball results

Responses in the auditory oddball task were evaluated relative to tone onset and across the full − 200 to + 600 ms epoch for standards and targets separately using continuous time-frequency ANOVAs. Group effects were examined for single-trial power (STP) and inter-trial coherence (ITC) across Low (3–17 Hz), Beta (18–32 Hz), and Gamma (33–55 Hz) ranges. Effects were summarized descriptively using group means and standard deviations from the statistically significant time-frequency regions. For DSM diagnosis comparisons, see Supplementary Results.

### Oddball Standards

#### Single-trial power (STP; response strength)

##### (5) Low-frequency STP (-200 to 600 ms).

Low-frequency power during standard stimulus trials showed robust and graded group differences spanning the entire epoch. BT2 exhibited the highest power (Δ = 0.64), whereas BT1 showed the lowest power (Δ = −0.74). BT3 (Δ = 0.22) and healthy participants were intermediate (Table 1). This produced a graded ordering of BT2 > BT3 > HC > BT1 that was stable across the entire epoch (Fig. 2A).

##### (6) Beta STP (-200 to 600 ms).

Beta-band power showed the largest group separations. BT2 exhibited the highest beta power (Δ = 0.70), whereas BT1 showed the lowest power (Δ = −0.95). BT3 (Δ = 0.16) and healthy participants were intermediate (Table 1). This graded ordering (BT2 > BT3 > HC > BT1) was present across the full epoch and represented the strongest separation between groups during standard stimulus processing (Fig. 2B).

##### (7) Gamma STP (-200 to 600 ms).

Gamma-band STP differences were smaller but followed a similar pattern as Beta STP. BT2 had the highest gamma power (Δ = 0.56), whereas BT1 showed the lowest power (Δ = −0.67). BT3 (Δ = −0.04) and healthy participants did not differ (Table 1). This produced an overall ordering of BT2 > (BT3 = HC) > BT1 across the epoch (Fig. 2C).

#### Inter-trial coherence (ITC; temporal precision)

##### (8) Low-frequency ITC (25–146 ms).

In the early post-stimulus interval, healthy participants had the highest phase consistency (Table 1), whereas BT2 had the lowest (Δ = −0.56). BT1 Δ = −0.34) and BT3 (Δ = −0.25) were intermediate, producing an ordering of HC > BT3 > BT1 > BT2 (Fig. 2D).

##### (9) Low-frequency ITC (205–320 ms).

A second low-frequency ITC cluster showed a similar ordering (Table 1). Healthy participants again had the highest coherence, followed by BT3 (Δ = −0.20), BT1 (Δ = −0.35), and BT2 (Δ = −0.48). This ordering (HC > BT3 > BT1 > BT2) was the same as the earlier low-frequency ITC epoch (Fig. 2D).

##### (10) Beta ITC (65–126 ms).

A significant beta-band ITC cluster occurred between 65 and 126 ms post-stimulus. Healthy participants had the highest coherence (M = 0.0468, SD = 0.0212), whereas BT2 had the lowest (M = 0.0376, SD = 0.0178, Δ = −0.43). BT1 (M = 0.0401, SD = 0.0189, Δ = −0.32) and BT3 (M = 0.0429, SD = 0.0197, Δ = −0.18) were intermediate. This produced an ordering of HC > BT3 > BT1 > BT2 (Fig. 2E).

##### (11) Gamma ITC (45–166 ms).

BT1 had the lowest phase consistency (Δ = −0.20), whereas BT2 (Δ = 0.01), BT3 (Δ = 0.04), and healthy participants did not significantly differ (Table 1 and Fig. 2F). A later gamma ITC cluster is visible in the time-frequency plots (480–600 ms), but this region did not significantly differentiate groups so was not considered in subsequent analyses.

### Oddball Targets

#### Single-trial power (STP; response strength)

##### (12) Low-frequency STP (-200 to 600 ms).

**Figure 3:**
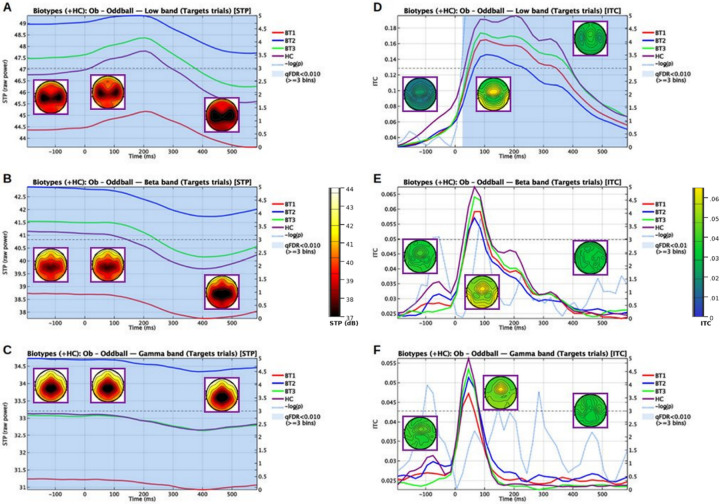
Oddball Target trials raw single-trial power (Left Column, A-C) and inter-trial coherence (Right Column, D-F). Group-average waveforms (~3–55 Hz) for Biotypes (BT1–BT3) and healthy participants (HC) across the −200 to 600 ms epoch. Shaded regions indicate time bins surviving cluster-corrected ANOVA thresholds (qFDR < .01, ≥3 contiguous bins); the dotted trace shows −log10(p) values across time. Whole-scalp averaging across 64 electrodes was employed to maximize signal reliability and isolate global response dimensions. Topographic maps (HC) at −200 to 0, 0 to 150, and 350 to 600 ms illustrate the scalp distributions underlying the averaged signal; biotype-specific topographies are provided in the Supplementary Materials.

Low-frequency power during target stimuli trials showed robust and graded group differences spanning the entire epoch. BT2 had the highest power (Δ = 0.66), whereas BT1 had the lowest power (Δ = −0.77). BT3 (Δ = 0.22) and healthy participants were intermediate (Table 1). This produced a graded ordering of BT2 > BT3 > HC > BT1 that was stable across the epoch (Fig. 3A).

##### (13) Beta STP (-200 to 600 ms).

Beta-band target-related responses showed the largest group separation and followed the same graded pattern (Table 1). BT2 had the highest beta power (Δ = 0.77), and BT1 had the lowest power (Δ = −0.91). BT3 (Δ = 0.19) and healthy participants were intermediate. This ordering (BT2 > BT3 > HC > BT1) was consistent across the entire epoch (Fig. 3B).

##### (14) Gamma STP (-200 to 600 ms).

Gamma-band STP differences were smaller in magnitude but followed a similar graded pattern to Beta STP (Table 1). BT2 had the highest gamma power (Δ = 0.58), whereas BT1 had the lowest power (Δ = −0.64). BT3 (Δ = −0.01) and healthy participants did not differ. This produced an overall ordering of BT2 > (BT3 = HC) > BT1 (Fig. 3C).

#### Inter-trial coherence (ITC; temporal precision)

##### (15) Low-frequency ITC (25–600 ms).

In response to target stimuli, low-frequency ITC showed clear and temporally circumscribed group differences (Table 1). Healthy participants had the greatest phase consistency, whereas BT2 had the lowest (Δ = −0.77). BT1 (Δ = −0.46) and BT3 (Δ = −0.23) were intermediate, producing an ordering of HC > BT3 > BT1 > BT2 (Fig. 3D).

##### Beta ITC.

Analyses of beta-band ITC in response to targets did not reveal statistically significant group differences (Fig. 3E).

##### Gamma ITC.

Gamma-band ITC likewise showed no statistically significant group differences, with no regions exceeding threshold in the ANOVA plots (Fig. 3F).

### Linear discriminant analyses

Linear discriminant analysis (BT1, BT2, BT3, HC) was performed using 15 differentiating STP and ITC features derived from paired-stimuli and oddball paradigms (For DSM diagnosis analyses, see DSM Supplementary Results). The overall discriminant model was significant (Wilks’ Λ = .546, χ^2^(45) = 1288.55, p < .001). Sequential tests indicated that only Function 2 provided significant additional discrimination beyond Function 1 (Wilks’ Λ = .850, χ^2^(28) = 347.54, p < .001). Function 1 accounted for 76.1% of group discrimination variance (eigenvalue = .555; canonical r = .597), and Function 2 accounted for an additional 21.4% (eigenvalue = .156; canonical r = .367).

**Figure 4:**
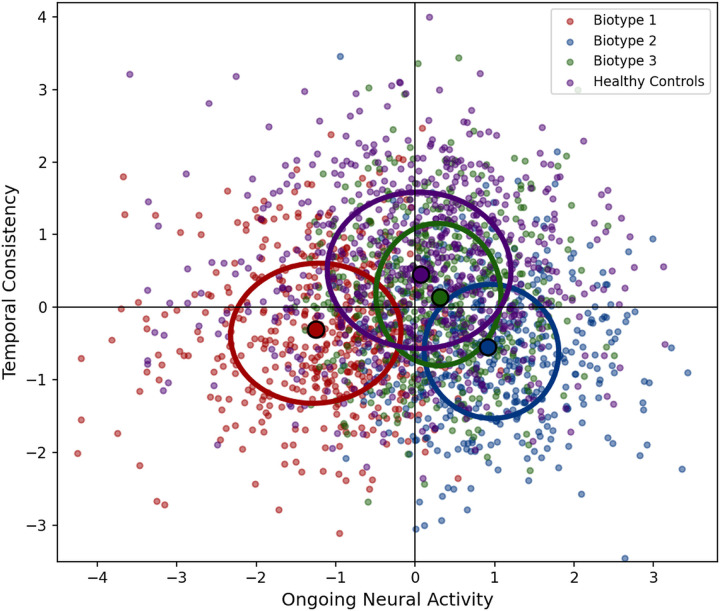
Linear discriminant analysis of EEG time–frequency features across psychosis Biotypes and healthy controls. Each point represents one subject positioned according to their overall EEG response profile, with axes reflecting weighted combinations of neural features that significantly differentiate groups along Function 1 and Function 2 (ps < .001). Large, filled circles mark group centroids, and ellipses indicate one standard deviation of each group distribution, illustrating central tendency and variability.

Inspection of the canonical loadings indicated that Function 1 was dominated by epoch-spanning beta-band STP across both paradigms, including paired-stimuli (−200 to 880 ms; r = .91), oddball targets (−200 to 600 ms; r = .81), and oddball standards (−200 to 600 ms; r = .80), as well as substantial contributions from low-frequency STP in paired-stimuli (r = .75), oddball targets (r = .67), and oddball standards (r = .66) (Table 1). These loadings indicate that Function 1 reflects a dimension of **ongoing neural activity**, with beta-band power serving as the dominant contributor. Standardized group centroids along Function 1 showed a graded ordering, with BT1 strongly negative (-1.25), BT2 strongly positive (0.92), and BT3 (0.31) and healthy participants (0.07) occupying intermediate positions (Fig. 4). An ANOVA confirmed a significant effect of Biotype on **ongoing neural activity**, F(3, 2138) = 405.84, p < .001, η^2^ = .363. Bonferroni-adjusted pairwise comparisons revealed that all groups differed significantly (all ps ≤ .001), with the ordering BT1 < < HC < BT3 < < BT2 (Fig. 4).

Function 2 was defined primarily by low-frequency ITC features reflecting across-trial **temporal consistency** of responses. The strongest contributions originated from paired-stimuli ITC spanning approximately 5–665 ms post-stimulus (r = .70), oddball target ITC across 25–600 ms (r = .74), and temporally circumscribed oddball standard ITC windows (25–146 ms, r = .55; 205–320 ms, r = .52) (Table 1). Standardized group centroids on **temporal consistency** showed healthy participants were most positive (0.45) and BT2 most negative (− 0.55), while BT3 (0.14) and BT1 (-0.30) were intermediate (Fig. 4). An ANOVA revealed a significant effect of Biotype on **temporal consistency**, F(3, 2138) = 142.42, p < .001, η^2^ = .167. Bonferroni-adjusted comparisons again showed that all groups differed significantly (all ps ≤ .001), with the ordering HC > BT3 > BT1 > BT2 (Fig. 4).

The Pythagorean theorem was used to quantify group separations in the 2D space of Fig. 4. The standardized differentiation of BT1 from healthy was 1.52, differentiation of BT2 from healthy was 1.31, and differentiation of BT3 from healthy was 0.39. The difference between BT1 and BT2 was 2.18, the difference between BT1 and BT3 was 1.62, and the difference between BT2 and BT3 was 0.92. In comparison, DSM Euclidean centroid distances were largest between SZ and BP (d = 0.45) and SZ and HC (d = 0.36), intermediate between SADP and BP (d = 0.35) and SADP and HC (d = 0.28), and smallest between BP and HC (d = 0.26) and SZ and SADP (d = 0.10). See Supplementary results for complete information.

## Discussion

The current investigation probed oscillatory features of single trials underlying paired-stimuli and oddball auditory EEG paradigms in psychosis. We predicted that (i) STP would emerge as an independent response magnitude dimension on which BT1 and BT2 would be at opposite ends of the spectrum, (ii) ITC would discriminate Biotype groups on a separate temporal precision dimension, (iii) a combination of these dimensions would more effectively discriminate Biotype groups than either measure alone, and (iv) DSM psychosis diagnoses would not benefit from separating response strength from response consistency. Results were consistent with these four predictions, with implications for understanding neurophysiological heterogeneity in idiopathic psychosis. Two discriminating dimensions emerged: (i) ongoing neural activity captured by low and beta frequency STP (76.1% of discrimination variance), reflecting background cortical excitability rather than stimulus-evoked response, and (ii) temporal consistency captured by low-frequency (3–17 Hz) ITC in distinct post-stimulus windows (21.4% of discrimination variance). The joint use of those dimensions more effectively differentiated Biotypes than either measure alone. Differences between DSM diagnoses were not obviously improved by STP and ITC. Each of these findings is addressed below.

Intrinsic neural activity unbound to stimulus processing is a dominant discriminating feature within idiopathic psychosis [28, 29]. This is true even after accounting for general cognitive performance, which covaries with most psychosis-related laboratory measures [12]. In this report, ongoing neural activity observed during stimulus processing worked similarly [7, 10, 35]. Background brain activity is a consistent, stable, and prominent neural feature of idiopathic psychosis [1, 3, 10]. Analysis of single trials clarified that these neural activity differences (BT2 > > BT3 > HC > > BT1) are enhanced in low and beta frequencies. An additional outcome of this report was that temporal consistency of lower frequency neural oscillations also discriminates idiopathic psychosis. Unlike ongoing neural activity, temporal consistency of neural oscillations is bound to stimulus processing. Nevertheless, this dimension did not mirror ERP amplitude differences of B-SNIP Biotypes (BT2 > HC=BT3 > > BT1) but showed that temporal consistency is highest in healthy persons and lowest in BT2 (HC > BT3 > BT1 > BT2).

The deviations in ongoing neural activity that characterize BT1 and BT2 span at least theta, alpha, and beta frequency ranges. Power across those bands is often associated with different cognitive functions of relevance to psychosis [36, 37, 38]. The consistency of BT1 and BT2 differences over a wide frequency range, and seemingly independent of stimulus processing, suggests a common neurophysiological cause. Likewise, temporally consistent lower frequency oscillations, primarily in the theta and alpha ranges, support long-range neural communication across distributed neural networks [39]. Disruptions in this synchrony can alter top-down predictive signals, adequacy of bottom-up sensory input, and compromise cognitive functions like stimulus evaluation, context updating, and cognitive control [40, 41].

ERP patterns and amplitudes observed in other publications [10] do not mirror these STP and ITC patterns. Moratti et al. [42], using steady-state stimuli where ERP-STP-ITC comparisons are straightforward, showed that in healthy persons ITC accounts for the overwhelming majority of ERP variance (with STP a distant second). The present findings illustrate that relationships between ERPs, STP and ITC may differ between B-SNIP Biotypes and between psychosis Biotypes and the healthy group. This fact diminishes the value of ERPs alone as probes for understanding etiological mechanisms within idiopathic psychosis. It also offers opportunities for testing specific theories of etiopathology conflated and complicated by using DSM psychosis diagnoses as targets.

For instance, BT2’s pattern may be accounted for by disinhibition secondary to faulty inter-neuronal performance in cortical networks [43]. There are multiple inhibitory neurotransmitters of possible relevance, but GABA receives considerable attention in psychosis. GABAergic neurons shape oscillatory power across multiple frequency ranges [44, 45], with GABA levels specifically predicting theta-alpha and beta oscillatory activity in schizophrenia [46]. BT2’s elevated STP is consistent with cortical disinhibition from GLU/GABA imbalance [54]. Dysfunction in somatostatin-positive interneurons is specifically implicated in theta, alpha, and beta dysregulation through impaired parvalbumin-positive interneuron gating [47]. This simultaneously elevates broadband oscillatory power and degrades trial-to-trial phase similarity, consistent with elevated resting beta in schizophrenia and their first-degree relatives [48]. A disinhibition mechanism is consistent with BT2’s poorer inhibitory control in sensorimotor tasks [3].

Alternatively, BT1’s depressed STP is consistent with insufficient excitatory drive of cortical pyramidal neurons. Although there are a few possible mechanisms of relevance, Javitt [16] proposed the BT1 pattern was secondary to NMDA receptor hypofunction. NMDA receptor antagonists like PCP and ketamine, which can induce psychosis-like clinical features [49] can yield electrophysiology patterns reminiscent of BT1 in so-called cortical ensembles [43, 50, 51]. These patterns and the known action of clozapine on electrophysiology provided the foundation of an ongoing clinical trial proposing that BT1 will preferentially benefit from this second-generation antipsychotic medication [2].

A few issues should be considered when evaluating these outcomes. First, B-SNIP has consistently demonstrated limited medication associations with EEG/ERP biomarkers [3, 10]. The same is true here, but post-hoc analyses are not an optimal strategy for testing such associations. Nevertheless, most psychosis participants were receiving psychopharmacology consistent with their clinical diagnosis. The fact that those DSM diagnoses are spread across B-SNIP Biotypes, especially BT1 and BT2, argues against the possibility of systematic medication confounds. Second, STP and ITC were computed as grand averages across 64 sensors. This was justified by across sensor ICCs being high for Low and Beta STP (.59 to .79) and moderate for Low and Beta ITC (.25 to .53). Both STP (.47 to .52) and ITC (.19 to .28) reliabilities were worse for gamma activity. Regardless, reliabilities adjusted for number of trials were high for all conditions (.936–.996; Supplementary Results). Third, gamma is high frequency oscillations of low amplitude useful for local (short range) neuronal communication (a cycle lasts only 25 msec at 40 Hz). It is more sensitive to poor signal-to-noise and low spatial sampling. The modest contribution of gamma activity in relation to lower frequencies does not necessarily mean it is less important for understanding idiopathic psychosis. Using more dense sensor arrays and combining EEG and MEG in subsequent investigations may be useful for more accurately describing gamma band contributions. Fourth, like other B-SNIP investigations, BT3 showed minimal deviations from healthy neural function, especially in relation to BT1 and BT2. BT3 do show modest deviations in registering stimulus salience [10] and modest reductions in lateral thalamic nuclei volume [12] after accounting for the larger and more dramatic effects of cognitive performance and neurophysiology that define BT1 and BT2. Perhaps we have yet to discover the neurophysiological features central to BT3 etiology. It is also possible BT3 come by their psychosis symptomatology via an entirely different path from BT1 and BT2, as happens within other medical disciplines [52, 53]. Finally, understanding of DSM diagnoses is not obviously improved by ERPs [3, 10] or STP and ITC quantifications. Effects sizes for the measures even between the most extreme DSM groups are less than 0.5 standard deviation units. This either means that direct measures of brain electrophysiology are not useful for the study of idiopathic psychosis (which seems inconsistent with a mountain of evidence), or DSM psychosis diagnoses are not useful for electrophysiological investigations.

## Supplementary Material

Supplementary Files

This is a list of supplementary files associated with this preprint. Click to download.

- DSMSupplementaryResultsGRECOMP.docx
- SUPPLEMENTARYMETHODSGRECOMP.docx
- SupplementalResultsGRECOMP.docx

## Figures and Tables

**Table 1:** Structure Matrix Showing Correlations Between EEG Features and Canonical Discriminant Functions

Task	Band	Measure	Time window (ms)	Function 1	Function 2	M (SD)			
						BT1	BT2	BT3	HC
Paired stimuli	Beta	STP	−200 to 880	.910*	−.226	38.23 (2.37)	42.77 (1.96)	41.22 (1.85)	40.54 (2.53)
Oddball targets	Beta	STP	−200 to 600	.812*	−.167	38.30 (2.45)	42.35 (2.08)	40.95 (1.88)	40.50 (2.41)
Oddball standards	Beta	STP	−200 to 600	.804*	−.113	38.70 (2.49)	42.82 (2.18)	41.49 (1.97)	41.08 (2.50)
Paired stimuli	Low	STP	−200 to 880	.750*	−.200	44.14 (3.18)	49.30 (2.86)	47.67 (2.59)	46.65 (3.22)
Oddball targets	Low	STP	−200 to 600	.672*	−.110	44.44 (3.04)	48.72 (2.98)	47.42 (2.37)	46.75 (3.00)
Oddball standards	Low	STP	−200 to 600	.659*	−.127	44.52 (3.23)	49.03 (3.15)	47.68 (2.66)	46.95 (3.27)
Oddball standards	Gamma	STP	−200 to 600	.524	−.133	31.22 (3.27)	34.71 (2.89)	33.02 (2.64)	33.12 (2.83)
Oddball targets	Gamma	STP	−200 to 600	.517	−.148	31.11 (3.26)	34.56 (2.91)	32.91 (2.64)	32.93 (2.83)
Paired stimuli	Gamma	STP	−200 to 880	.557	−.238	30.69 (3.33)	34.48 (2.69)	32.62 (2.53)	32.50 (2.81)
Oddball targets	Low	ITC	25 to 600	−.061	.741*	0.12 (0.05)	0.10 (0.04)	0.13 (0.05)	0.14 (0.05)
Paired stimuli	Low	ITC	5 to 665	−.017	.696*	0.12 (0.05)	0.10 (0.05)	0.13 (0.06)	0.14 (0.06)
Oddball standards	Low	ITC	25 to 146	−.062	.547*	0.15 (0.07)	0.13 (0.06)	0.15 (0.07)	0.17 (0.08)
Oddball standards	Low	ITC	205 to 320	−.035	.518*	0.09 (0.05)	0.08 (0.05)	0.10 (0.05)	0.11 (0.06)
Oddball standards	Beta	ITC	65 to 126	.018	.316	0.05 (0.03)	0.05 (0.03)	0.06 (0.03)	0.06 (0.04)
Oddball standards	Gamma	ITC	45 to 166	.102	.081	0.03 (0.02)	0.03 (0.02)	0.03 (0.02)	0.03 (0.02)

Abbreviations: STP = single-trial power; ITC = inter-trial coherence; BT = Biotype; HC = healthy controls. Asterisks (*) denote the function with the largest absolute loading for each feature.

## Data Availability

To request access to B-SNIP data used in this manuscript, visit https://nda.nih.gov/. There is a “Get Data” tab at the top of the main page, under which is found a “Request Data Access” link. Request psychosis and healthy subject data, and the below described biomarker data, from: B-SNIP1 (https://nda.nih.gov/edit_collection.html?id=2274). B-SNIP2 (https://nda.nih.gov/edit_collection.html?id=2165). PARDIP (https://nda.nih.gov/edit_collection.html?id=2126).
